# Pediatric Acute Mastoiditis: Our Experience in a Tertiary Care Center

**DOI:** 10.7759/cureus.15052

**Published:** 2021-05-16

**Authors:** Inku B Shrestha, Monika Pokharel, Ashish Dhakal, Aakash Mishra

**Affiliations:** 1 Department of Ear, Nose and Throat - Head and Neck Surgery, Kathmandu Medical College Teaching Hospital, Kathmandu, NPL; 2 Department of Ear, Nose and Throat - Head and Neck Surgery, Kathmandu University School of Medical Sciences, Dhulikhel, NPL

**Keywords:** acute mastoiditis, complications, management, nepal, pediatrics

## Abstract

Introduction

Acute mastoiditis (AM) is a common intra-temporal complication of acute otitis media (AOM) and is more commonly seen in children. Occasionally, it presents as the first sign of ear disease. This study aimed to evaluate the clinical course of AM and determine therapeutic options for pediatric patients presenting with AM.

Methods

This was a prospective, observational study conducted on patients with AM presenting at a tertiary center during one year period. Convenience sampling was employed and 79 pediatric patients (18 years or below) were recruited for the study. Data on the demographic profile of patients, the treatment offered, duration of hospital stay, and outcome were analyzed.

Result

In our study, 62% were male patients (n = 49) and 38% (n = 30) were females. The mean age of patients was 9.32 ± 5.3 years and a history of AOM was present in 60 (75.9%). On admission, the most common presentation was post-auricular inflammation (100%) followed by otalgia (79.7%), fever (59.5%), aural protrusion (54.4%), and otorrhoea (51.9%). Culture reports were available for 54 (68.4%) patients and 30 (38%) grew organisms. The cultured organisms were *Streptococcus pneumonia* (20.3%), *Pseudomonas aeruginosa* (10.1%), *Streptococcus pyogenes* (3.8%), and *Staphylococcus aureus* (3.8%). Most patients were managed conservatively (n = 66, 83.5%) whereas surgery was performed in 16.5% (n = 13) patients. The mean hospital stay was 5.58 ± 1.99 days. The need for surgical management was significantly associated with age >5 years (*p* = 0.006), history of AOM (*p* = 0.026) and the presence of complications (*p* = 0.012). Subperiosteal abscess (SA) was present in 21 (26.6%) patients and one had facial palsy. SA along with AM had a mean hospital stay of 8.5 ± 0.77 days compared to 4.94 ± 1.43 days in case of isolated AM (*p *< 0.001) and the mean age of presentation in SA with AM was 11.97 ± 5.13 years compared to 8.29 ± 5.14 years in case of isolated AM (*p = *0.006). All patients recovered and were followed up to three months with no recurrence, complications, or sequelae.

Conclusion

Most of the cases of acute mastoiditis follow previous AOM episodes. With early recognition and effective treatment, the prognosis is good.

## Introduction

Acute mastoiditis (AM) is likely to develop as a complication of acute otitis media (AOM) [1-3]. AM is the most common and feared complication of AOM, which causes serious and life-threatening complications beyond the temporal bone, including meningitis, epidural, and subdural abscess, brain abscess, and lateral sinus thrombophlebitis [4].

The incidence of AM in the pediatric age group is increasing [5], even more in developed countries [1,6,7]. A similar tendency has been observed for suppurative intracranial complications of AM [8]. Abuse of or inadequacy of antibiotic treatment has been attributed to such phenomenon [6,9].

The management protocols vary between different studies. Some centers have adopted a rather conservative method for its management and its associated complications [10,11]. Even minor surgical interventions, such as myringotomy, have been questioned since mere parenteral antibiotics are considered adequate for the treatment of AM [12-15]. Such a conservative approach may be sufficient for some patients but not all [3].

The objective of this study was to discuss our experiences in the diagnosis and treatment of pediatric mastoiditis and their outcome.

## Materials and methods

This was a prospective, observational study conducted on 79 patients, 18 years or younger, who presented to the Department of ENT, Dhulikhel Hospital, from February 2017 to February 2018. A convenience sampling method was used. Written informed consent was obtained from family members/relatives of patients and confidentiality of information was maintained. Patients with AM were involved in the study and diagnosis was based on patients history, laboratory parameters, and clinical and otomicroscopic examinations. AM was diagnosed when one or more of the physical signs of mastoiditis (swelling, erythema, tenderness of retro-auricular region, and anteroinferior displacement of the auricle) were present with concomitant or recent (≤4 weeks) AOM [13]. In the absence of AOM, the presence of two or more signs of retro auricular inflammation diagnosed AM. Patients, 18 years or younger, with a diagnosis of AOM based on clinical signs and symptoms and providing consent, were included in the study. Patients above 18 years of age, refusing admission, presenting with chronic otitis media with cholesteatoma, otitis externa, or patients who lost for follow-up were excluded from the study.

Patients’ characteristics such as demographic information, site of infection, clinical symptoms and signs, previous history of AOM, laboratory tests, radiological and microbiological report, complications, treatment received, duration of hospital stay, and outcome were noted. Children under five years of age were classified as younger children and children of five years or above as older children. The laboratory values of white blood cell (WBC) count and differential count were recorded. Culture specimens were obtained from the pus draining spontaneously in the external auditory canal or after tympanocentesis. In cases where drainage of abscess or mastoidectomy was done, swabs of pus were taken from the mastoid cavities using sterile ear swabs and sent to clinical microbiology laboratory in sterile transport medium. Radiological tests such as high resolution computed tomogram (HRCT) of temporal bone were done in patients with signs of impending complications or those with no improvement after beginning conservative management. In cases where CT scan was not affordable to patients, a plain X-ray of mastoid was done. The radiological findings of interest were degrees of pneumatization of mastoid, content in middle ear cleft, possible bony destruction, or spread of infection beyond temporal bone.

Patients were divided into two groups based on treatment. Conservative group refers to management with injectable antibiotics and myringotomy or incision and drainage. The operative group included the patients requiring mastoidectomy along with injectable antibiotics and myringotomy or incision and drainage. All patients received broad-spectrum empiric intravenous antibiotics shortly after admission based on the presumed presence of major bacterial pathogens. The combination therapy consisted of a third-generation cephalosporin (Ceftriaxone sodium in a dose of 20-50mg/kg body weight) with or without Metronidazole (7.5mg/kg, every six hourly). Antipyretics and analgesics were given on an individual basis. All children were given oral antibiotics for another 5-7 days after discharge. Myringotomy when done was performed in the posteroinferior quadrant and the middle ear was drained. Ventilation tubes were not inserted into our patients. Patients were followed up in one week, two weeks, and three months. Recurrence after three months was considered a new episode of AM. Failure of treatment was defined by persistence of post-auricular pain, and /or fever.

Descriptive data were presented as frequency, minimum, maximum, mean, and standard deviation values. Statistical package for the social sciences (SPSS) 22.0 software (SPSS Inc, Chicago, IL) was used for data analysis. Chi-square test and two-tailed paired t-test were used to analyze data collected. For all statistical analyses, significance was accepted at a p-value less than 0.05.

## Results

Patient characteristics

**Figure 1:**
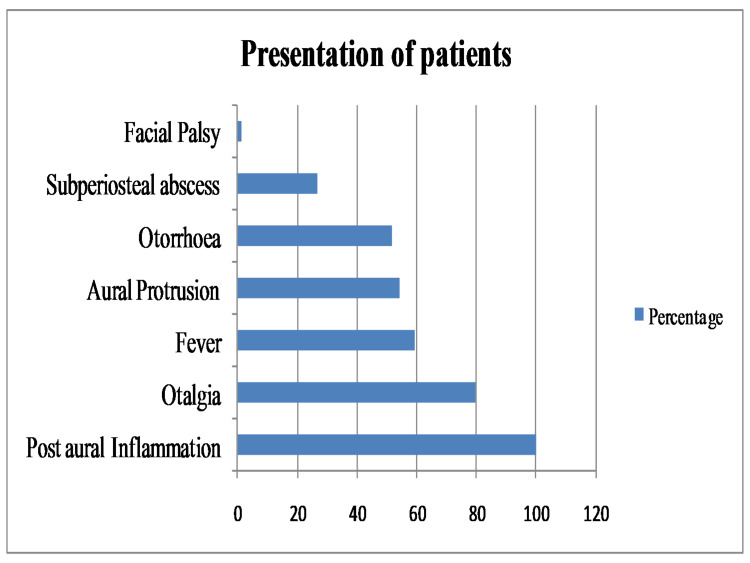
Signs and symptoms of patients

A total of 79 patients participated in the study with a mean age of 9.32 ± 5.33 years (one to 18 years). The male to female ratio was 1.6:1, 49 (62%) children were male and 30 (38%) were female. There were 26 (32.9%) children ≤5 years and remaining children between five and 18 years of age. History of AOM ≤4 weeks was present in 60 (75.9%) children. All cases had unilateral involvement and the right ear was involved in 50.6%. On admission, all children had some evidence of post-auricular inflammation (redness and/or swelling and/or tenderness), and most of them presented with otalgia (79.7%). Significant fever (temperature > 38°C) was the second most common presentation followed by aural protrusion and active ipsilateral otorrhoea as shown in Figure 1.

Among the 54 (68.4%) pus samples that were sent for culture reports, only 30 grew identifiable bacteria. *Steptococcus pneumonia* was the most common organism followed by *Pseudomonas aeruginosa, Streptococcus pyogenes,* and *Staphylococcus aureus*. Leucocytosis (WBC count > 15,000/cubic mm) was found in 51 (64.6%) cases. A plain high-resolution CT scan of the temporal bone was done in 13 patients and a plain mastoid X-ray in eight patients. Complication, such as subperiosteal abscess (SA), was found in 21 (26.6%) patients while one case had facial nerve palsy associated.

Management of acute mastoiditis

Conservative treatment was given in 66 (83.5%) of patients and antibiotics alone were sufficient to treat 41 (51.9%) patients. Antibiotics with drainage of pus were required in 25 (31.6%) cases. Simple mastoidectomy was performed in 13 (16.5%) children out of which seven children were uncomplicated cases of AM but showed poor response to the initial conservative approach (Table 1). All patients with age ≤5 years were managed conservatively and children of age >5 years were more likely to undergo surgical management (p = 0.006). The decision for surgery was made on these patients between the third and fifth day of admission. The presence of complications at initial presentation (p = 0.012) and history of recent AOM (p = 0.026) were associated with the need for surgical management. The mean duration of hospital stay for conservative management was 4.94 ± 1.43 days. No postoperative complications were noted in patients undergoing simple mastoidectomy.

**Table 1:** Comparison between the conservative management and surgical management *Refers to a significant result; AOM: Acute otitis media

Variables	Treatment		Total	p-value
	Conservative (n = 66)	Surgical (n = 13)		
Age				
≤5 years	26	0	26	0.006*
>5years	40	13	53	
History of AOM				
Yes	47	13	60	0.026*
No	19	0	19	
Complications				
No complication	51	6	57	0.012*
Subperiosteal abscess	15	6	21	
Facial nerve palsy	0	1	1	
Organisms				
Steptococcus pneumoniae	14	2	16	0.327
Pseudomonas aeruginosa	5	3	8	
Streptococcus pyogenes	2	1	3	
Staphylococcus aureus	2	1	3	

Management of acute mastoiditis with complications

The mean age of presentation of AM with SA was 11.97 ± 5.13 years compared to 8.29 ± 5.14 years in the case of isolated AM (p = 0.006). Initial treatment in children with SA included abscess drainage along with antibiotics (n = 15) or simple mastoidectomy (n = 6). Bony erosion concomitant with abscess formation was observed in children undergoing mastoidectomy. One case of AM with facial palsy was treated with surgery along with antibiotics and recovered. Patients with SA were discharged from the hospital after a mean duration of 8.5 ± 0.77 days. Hospital stay for AM with SA was associated with a longer duration of hospital stay (p < 0.001) (Table 2).

**Table 2:** Comparison between acute mastoiditis and acute mastoiditis with subperiosteal abscess *Refers to a significant result; AOM: Acute otitis media

Variables	Acute mastoiditis (n= 57)	Acute Mastoiditis with Subperiosteal abscess (n=21 )	p-value	
Age ( Mean ± SD)	8.29 ± 5.14 years	11.97 ± 5.13 years	0.006*	
Sex				
Male	35	14	0.67	
Female	22	7		
History of recent AOM				
Yes	40	19	0.064	
No	17	2		
Duration of hospital stay	4.94 ± 1.43 days	8.5 ± 0.77 days	<0.001*	
				Management				
Conservative	51	15	0.05	
Surgery	6	6		

Outcome of patients

All the children were cured and discharged after the treatment. During our study period, except for those complications present on admission, no additional intracranial or intratemporal complications related to the further spread of the disease developed in these patients. Similarly, on follow-up of patients after seven days, 15 days, and three months of discharge, no long-term intratemporal or intracranial complications, sequelae, or cases of “masked” mastoiditis or any recurrence were found.

## Discussion

AM most commonly results as a complication of acute inflammation of the middle ear. It mainly affects the pediatric population [1,2,5]. Diagnosis is based on clinical signs of postauricular swelling, erythema, tenderness, and forward protrusion of the auricle. Otomicroscopic examination is a very powerful tool in making an early diagnosis of AM which helps in initiating therapy. AM may be overlooked in developing countries like Nepal where health care services and experienced doctors may not be available everywhere. Thus, its incidence remains a burden. In the context of Nepal, very limited data have been published on mastoiditis among children.

Our results showed, 32.9% were younger children <5 years, and 67.1% were >5 years. It could be because young children are least immunologically prepared to fight highly virulent organisms and diagnosis of AOM is difficult to make due to the narrow external auditory canal which is often obstructed with wax. Moreover, the possibility of hematogenous spread of infection without underlying otitis media has also been postulated in literature [1,2].

*S. pneumonia* was the most dominant organism grown in our study. However, failure to isolate bacteria in all the samples could be due to early initiation of antibiotic treatment before culture reports or delay in processing the sample [3,16]. Blood tests, such as leucocytosis, elevated erythrocyte sedimentation rate (ESR), and C-reactive protein (CRP) levels, were done to support the diagnosis of acute inflammation. Although these parameters may provide information about disease progression or treatment effectiveness, they are not prognostic parameters for disease outcome.

Computed tomography (CT) Imaging was performed only in 13 children with suspected intracranial complications, recurrent AM, atypical presentations with doubtful diagnosis, or in the absence of favorable response to initial therapy. Mastoid bone radiographs were obtained in eight cases where the patient could not afford a CT scan. Erosion of mastoid can be seen well with a CT scan. Radiological investigations of small children are used with caution due to the need for general anesthesia and the risks involved with irradiation [2,3]. In developing countries like ours, it is also a luxury only afforded by the wealthy. Therefore, unless serious complications are suspected clinically, they are not typically employed.

SA, as a complication of AM, was found in 26.6% of cases. Cases of SA were treated with conservative treatment such as retro-auricular needle aspiration and /or incision, while 13 cases had to undergo mastoidectomy. Children should be under cautious clinical monitoring in all cases. In patients lacking clinical improvement, mastoidectomy should be performed within 24-48 hours. Other authors have also described conservative management of SA and simple mastoidectomy can be performed in non-responsive cases [2,3,10,12-15]. The treatment of SA has been long debated, but we followed the motto “Ubi pus ibi evacua” (where you find pus, remove it).

The group treated with mastoidectomy had a longer hospital stay, which is similar to a study performed on 115 Swedish patients [17]. Immediate surgical treatment including a neurosurgical procedure is indicated for intracranial complications, to clear the source of infection. It can be performed in the same setting or delayed until the neurological conditions have stabilized [2]. 

In our study, a complication of left-sided lower motor neuron type facial palsy was found in a girl of 12 years of age with AM. She underwent cortical mastoidectomy along with antibiotics. The facial palsy completely recovered. There has been a reported case of three-month-old infant with facial palsy complicated by masked otomastoiditis [18]. Therefore, we would like to emphasize that otomastoiditis must be considered in the differential diagnosis of children with facial palsy. In uncomplicated cases, medical treatment alone led to complete recovery in most cases.

If possible, patients with mastoiditis should be managed in the hospital setting. In our study, all cases were admitted to the hospital and the choice of treatment was based on clinical, laboratory, and radiological findings. The most common bacteria which cause AM are *S. pneumoniae* and *P. aeruginosa*, so antibiotics are directed towards both gram positive and gram negative microorganisms. 

Mastoidectomy was the mainstay of treatment for AM in the pre-antibiotic era. At present, the availability of antibiotics has radically changed the management of the disease, and intravenous antibiotics, along with drainage of pus are effective in the management of uncomplicated cases of AM as reported in many studies given in Table 3.

**Table 3:** Mastoidectomy rates in retrospective series of pediatric acute mastoiditis

Author	Number of Cases	Mastoidectomy Procedure	Percentage (%)
Psarommatis IM et al. [3]	65	12	42
Gliklich et al. [4]	124	67	54
Ghaffar et al. [5]	57	19	33
Cohen – Kerem et al. [10]	44	4	11
Vassbotn et al. [11]	57	50	88
Spartley et al. [19]	49	12	24
Harley et al. [20]	58	21	39
Kvestad et al. [21]	38	13	34
Zapalac et al. [22]	75	29	39
Rosen et al. [23]	69	22	32
Vera-Cruz et al. [24]	62	11	18
Current study	79	13	16.5

The limitations of this study are a small number of patients and a short duration of the study. CT scan could not be done in all patients due to financial burden. Patients who refused admission due to financial or social issues could not be studied and the use of over-the-counter antibiotic therapy before admission could not be evaluated. This may have had a major effect on clinical course and microbiological study. Long-term follow-up was not possible due to geographic and financial conditions.

## Conclusions

Patients with AM can be identified early from the presentations of post-auricular inflammation. Use of antibiotics and drainage of abscess by retro-auricular needle aspiration and/or incision represent a simple and low-risk approach for management of AM and its complications. In our experience, we found that drainage of abscess with sufficient antibiotic coverage is safe as an initial conservative approach while simple mastoidectomy can be reserved for non-responding cases. With early recognition and effective treatment, the prognosis of AM is good.
